# Fatigue relief is possible

**DOI:** 10.3389/fnint.2026.1822424

**Published:** 2026-06-01

**Authors:** M. De Ventura, G. Mattioni, T. L’Abbate, M. Bertoli, J. Grifoni, G. Persichilli, A. Canelli, A. D’Onofrio, L. Paulon, F. Tecchio

**Affiliations:** 1 Laboratory of Electrophysiology for Translational Neuroscience (LET’S)—ISTC, CNR, Rome, Italy; 2 Department of Psychology, “Sapienza” University of Rome, Rome, Italy; 3 Italian Association of Multiple Sclerosis (AISM), Pescara, Italy; 4 Department of Neuroscience, Imaging and Clinical Sciences, University “G. D’Annunzio” of Chieti-Pescara, Chieti, Italy; 5 Faculty of Psychology, Uninettuno University, Rome, Italy; 6 Independent Researcher, Rome, Italy

**Keywords:** cognitive behavioral therapy, fatigue, mindfulness, physical activity, precision interventions, yoga

## Abstract

Chronic fatigue is widely discussed across clinical and non-clinical contexts, yet its definition and measurement remain heterogeneous and debated. In this fragmented landscape, adopting an efficacy-informed perspective grounded in mitigating interventions offers a reliable pathway to delineate the core features and regulatory mechanisms of chronic fatigue. This Mini Review aims to identify and synthesize interventions shown to be effective in mitigating chronic fatigue. A preliminary screening was conducted using the terms “fatigue” AND (“relief” OR “mitigation” OR “treatment”), thereby inherently focusing on persistent and functionally impairing forms of fatigue. The analysis was then restricted to the most cited evidence to highlight the most consolidated findings. Four classes of behavioral interventions consistently emerged as effective across heterogeneous conditions (stroke, cancer, multiple sclerosis, traumatic brain injury, and non-clinical contexts): cognitive behavioral therapy, mindfulness-based approaches, yoga, and physical activity. Their convergence suggests that chronic fatigue reflects, rather than condition-specific processes, the alteration of shared regulatory mechanisms, likely involving neurophysiological dynamics and associated biological markers, which can be further elucidated by integrating evidence from effective interventions with their neurophysiological and biomarker correlates. The evidence from this Mini Review on the transversal effectiveness of the same interventions supports leveraging their efficacy and the associated neurophysiological and biochemical changes to identify sensitive markers that track treatment response and inform the underlying mechanisms of fatigue. Future research should therefore prioritize standardized measures, shared markers, and personalized interventions, potentially supported by biochemical and neuromodulatory approaches.

## Introduction

Fatigue is an experience that everyone encounters. It typically functions as an adaptive signal, encouraging rest after sustained physical or cognitive effort. Nevertheless, it may become a persistent feeling of lack of energy, so intense and frequent that it hinders daily activities (Penner and Paul, 2017; Raizen et al., 2023). The International Classification of Diseases 11th Revision (ICD-11) defines fatigue (MG22) as “A feeling of exhaustion, lethargy, or decreased energy, usually experienced as a weakening or depletion of one’s physical or mental resource and characterised by a decreased capacity for work and reduced efficiency in responding to stimuli. Fatigue is normal following a period of exertion, mental or physical, but sometimes may occur in the absence of such exertion as a symptom of health conditions”. In its chronic form, fatigue may become a complex and disabling condition. Chronic fatigue syndrome can be considered an umbrella term for a multisystem disorder characterized by profound, persistent fatigue lasting more than 6 months and not alleviated by rest. It is often associated with additional symptoms, such as post-exertional malaise, cognitive dysfunction and sleep disturbances, and can emerge across different conditions, without a single identifiable cause. When persistent fatigue arises, it reflects a disruption in the dynamic relationship between the individual and their physical, social, and working environment.

Although chronic fatigue has been largely investigated in association with certain pathologies, such as cancer, stroke, multiple sclerosis, traumatic brain injury, post-acute sequelae of SARS-CoV-2 infection, myalgic encephalomyelitis/chronic fatigue syndrome, rheumatological disease, hearing loss and Parkinson’s disease (Raizen et al., 2023; Strauss et al., 2010; Trenado et al., 2023), it is a subjective experience that can impair health-related quality of life among people even without specific diagnosis. Recent data proves that chronic fatigue has an average prevalence of 7.7% among the general population worldwide, although frequency can be affected by multiple variables. Indeed, studies reported that women have higher levels of chronic fatigue than men, that lower socioeconomic status relates to higher levels of chronic fatigue and that the prevalence is higher in the Asian population rather than American and European ones (Dinos et al., 2009; Engberg et al., 2017; Yoon et al., 2023).

This Mini Review aims to identify and synthesize interventions shown to be effective in mitigating chronic fatigue.

## Evidence-based interventions for chronic fatigue mitigation

To investigate the existence of interventions effective in mitigating chronic fatigue, we executed a bibliographic search in Scopus, due to its extensive coverage of high-quality journals and the ability to rank results by citation count. A preliminary screening search was conducted to identify available interventions using the terms “fatigue” AND (“relief” OR “mitigation” OR “treatment”). The term “fatigue” was intentionally preferred over “chronic fatigue” due to the substantial heterogeneity in the definition and use of the latter. By coupling “fatigue” with intervention-related terms, the search inherently selected studies addressing persistent and functionally impairing forms of fatigue, thus capturing the chronic dimension of the symptom without relying on inconsistent terminology.

After identifying the interventions commonly employed for the treatment of chronic fatigue, a second search was performed using the terms “intervention name” AND “fatigue” (where “intervention name” refers to each intervention identified in the initial search). Results were then ranked by citation count, and an arbitrary threshold of 50 citations was applied to select the most established and widely adopted interventions for mitigating chronic fatigue across different conditions. Interventions not meeting this threshold were excluded. Finally, only studies specifically addressing chronic fatigue conditions, rather than fatigue in general (e.g., exertion-related fatigue in experienced athletes), were included.

These methodological choices were made with the awareness that they do not provide an exhaustive overview, but were intended to ensure the inclusion of robust and reliable evidence.

### Cognitive behavioral therapy

Cognitive Behavioral Therapy (CBT) is a form of psychotherapy that aims to identify and redirect negative thought patterns and behaviors.

Evidence claims that CBT is a promising treatment of chronic fatigue (Hofmann et al., 2012). This effectiveness is probably due to the fact that, as cognitive behavioral models suggest, a combination of physiological, behavioral, cognitive, and social factors contribute to fatigue. CBT aims to address the specific thinking patterns (e.g., catastrophic thoughts about the meaning of symptoms), behaviors (e.g., excessive avoidance or restriction of activity, or pushing oneself hard despite increasing fatigue) and emotion regulation strategies that are thought to interact with physiological processes that contribute to chronic fatigue symptoms (Rimes and Wingrove, 2013).

Studies found that CBT was more effective in the treatment of fatigued patients rather than the various control conditions examined, with a significantly greater decrease in chronic fatigue severity, and this result was found both in adults and adolescents (Deale et al., 1997; Prins et al., 2001; Stulemeijer et al., 2005).

A meta-analysis of 13 studies on individuals with chronic fatigue found a nearly medium mean effect size for CBT, with substantial heterogeneity across studies. This variability likely reflected differences in symptom severity, the intensity and methods of CBT treatments, and the measures used to assess chronic fatigue. Overall, these findings indicate that CBT yields promising results, although it does not consistently lead all patients to no longer meet criteria for chronic fatigue (Malouff et al., 2008).

Research on cancer, found that CBT was successful in mitigating chronic fatigue, significantly reducing it both after and during cancer treatment, more than the other interventions considered (Goedendorp et al., 2010; Poort et al., 2020). Researchers have also investigated the effects of Internet-based CBT interventions compared to usual care in breast cancer survivors, and found that participants in the treatment condition reported significantly less chronic fatigue, with a large effect size (Abrahams et al., 2017).

Chronic fatigue has been also investigated in multiple sclerosis. Van den Akker and colleagues conducted a systematic review on CBT effectiveness. They analyzed four studies and reported a moderately positive effect of CBT. However, they highlighted considerable heterogeneity in CBT protocols, a limitation commonly reported in meta-analyses and reviews on this topic (van den Akker et al., 2016).

It is also important to mention a quasi-experimental study in healthy people that aimed to assess the efficacy of a CBT-based one-day workshop intervention in a workplace setting for the reduction of work-related rumination and chronic fatigue. The results showed that individuals who attended the workshop reported significantly lower levels of chronic fatigue at follow-up. In line with the CBT model, it was hypothesized that participants were able to identify and challenge maladaptive cognitions in relation to their work, ultimately reducing perceived levels of chronic fatigue (Querstret et al., 2016).

### Mindfulness

Mindfulness is defined as the state of being openly aware of and attentive to what is taking place in the present, in one’s mind, body and surroundings. It is a component of consciousness believed to promote wellness through a disengagement from automatic habits and unhealthy behavior patterns, which leads to a clearer and more intense experience of reality. Scientific research indicates that mindfulness is associated with high self-esteem, optimism, life satisfaction and overall well-being (Brown and Ryan, 2003). Among mindfulness-based interventions, the two *mindfulness-based stress reduction* and *mindfulness-based cognitive therapy* protocols are the most used in chronic fatigue mitigation. They may operate through changes in repetitive negative thinking, cognitive and emotional reactivity, self-compassion and psychological flexibility to increase well-being (Gu et al., 2015).

Mindfulness-based interventions have proven their effectiveness in mitigating chronic fatigue in those who have tried CBT with little results (Rimes and Wingrove, 2013). Regarding cancer patients, promising research has shown that these interventions are effective in mitigating chronic fatigue and improving quality of life, alleviating distress and the fear of cancer recurrence (Park et al., 2020; Xie et al., 2020). Mindfulness-based interventions are also effective in mitigating chronic fatigue, mood disturbances, stress and in improving quality of sleep in cancer outpatients (Carlson and Garland, 2005).

In conclusion, a meta-analysis (257 subjects) suggested that mindfulness can be beneficial in alleviating chronic fatigue in patients with stroke, traumatic brain injury, and multiple sclerosis (Ulrichsen et al., 2016).

### Physical activity

Physical activity is defined as any bodily movement produced by the contraction of skeletal muscles (Caspersen et al., 1985), with WHO guidelines recommending at least two and a half hours per week at moderate intensity or one and a quarter hours at vigorous intensity (Bull et al., 2020). It has long been proven effective in maintaining well-being, having profound effects on multiple bodily systems (McEwen, 2007). Experiencing chronic fatigue is more common in sedentary compared to physically active people, since insufficient activity can partly express itself in fatigue. Notably, physical activity, recommended for preventing a range of somatic and psychiatric conditions, has also proven effective in alleviating chronic fatigue.

Although fatigued persons may be less motivated to engage in physical activity, since their condition has an impact on motivation and vitality, encouraging physical activity could be the solution to break the negative loop and mitigate chronic fatigue (Engberg et al., 2017; Puetz, 2006). A proposed approach in literature is Graded Exercise Therapy, which helps the participant to gradually return to physical activity and consequently reduce chronic fatigue. The intervention is tailored to the individual’s psychophysical needs, with exercise intensity increased through negotiation according to the participant’s perceived readiness. This approach has been suggested for people suffering from chronic fatigue, but literature is conflicting on this matter. In fact, while some authors encourage this treatment, others do not recommend physical activity, since in some cases it could increase post-exertional malaise, triggered by physical exertion (Raizen et al., 2023; White et al., 2023). This consideration underlines the importance of personalizing interventions for fatigued patients.

Clinical guidelines recommend physical activity for reducing chronic fatigue in cancer patients. In particular, aerobic exercises resulted in the improvement of both chronic fatigue and physical functioning (Frikkel et al., 2020; Kessels et al., 2018). Furthermore, a recent meta-analysis of 170 studies found that physical activity of diverse type (aerobic, neuromotor, resistance, combination) has an effect in mitigating chronic fatigue, regardless of the type of cancer, timing of intervention, modality (supervised or not) and setting (group or non-group) (Oberoi et al., 2018).

Physical activity is also effective in alleviating chronic fatigue in people affected by multiple sclerosis, as a recent meta-analysis of 31 articles shows (Razazian et al., 2020).

### Yoga

Yoga is an ancient Eastern tradition that originated in India. It generally consists of a combination of spiritual, moral, and physical practices, to help the individual attain self-awareness (Sadja and Mills, 2013). There are a variety of types of yoga but in the most common form adopted by Western society a yoga session typically includes the execution of physical postures (asanas), breathing techniques (pranayama), and meditation (dhyana), which cultivate more profound states of consciousness. Asanas may increase one’s physical flexibility, coordination, and strength, while pranayama aims to increase focus and relaxation and dhyana may calm and focus the mind, thus resulting in higher quality of life. Presumed benefits of yoga also include a sense of well-being, decreased pain, improved sleep quality, stress reduction, and control over physiological parameters (Büssing et al., 2012).

An early meta-analysis of 19 clinical studies (total *n* = 948), including participants with cancer, multiple sclerosis, undergoing dialysis, or affected by chronic pancreatitis, fibromyalgia, asthma, as well as healthy individuals, reported small positive effects of yoga on chronic fatigue, with GRADE recommendations indicating moderate confidence in the overall findings (Boehm et al., 2012). More recently, many studies have reported the effect of yoga on cancer patients. A meta-analysis of 17 studies evaluated the effects of yoga on mitigating chronic fatigue in people with breast cancer, finding that yoga had a small but statistically significant beneficial effect (Dong et al., 2019). Another study investigated the adjunctive effect of yoga in breast cancer survivors, comparing aerobic exercise training alone with the same program supplemented with yoga. Both groups showed improvements in chronic fatigue; however, integrating yoga resulted in a clear advantage as the combined intervention led to significantly greater benefits (Yaʇli et al., 2015). Moreover, studies found that greater practice and higher number of yoga classes were associated with higher chronic fatigue relief (Sadja and Mills, 2013).

Yoga ability to relieve chronic fatigue was also studied in people with mild to moderate multiple sclerosis. Comparing aerobic and yoga training in a parallel study design, chronic fatigue levels were significantly lower in both groups with no significant difference between them (Ahmadi et al., 2013).

## Discussion

### Converging behavioral pathways in chronic fatigue mitigation

**Figure 1:**
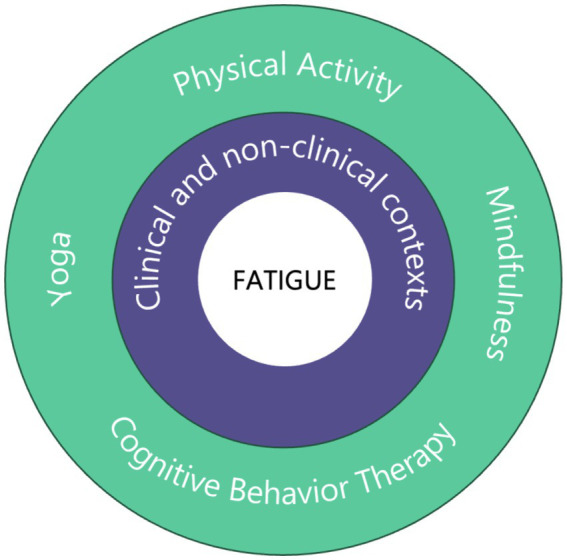
Converging behavioral pathways in chronic fatigue mitigation. The same four classes of interventions (external circle)—physical activity, yoga, cognitive behavioral therapy, and mindfulness—consistently reduce chronic fatigue across heterogeneous contexts (clinical and non-clinical conditions, including stroke, cancer, multiple sclerosis, traumatic brain injury, and healthy populations). Their convergence suggests that chronic fatigue reflects alteration of a shared regulatory mechanism.

A key and robust finding of this Mini Review is that the same four behavioral interventions—CBT, mindfulness-based approaches, yoga, and physical activity—consistently mitigate chronic fatigue across conditions, populations, and contexts. Moreover, individuals describe chronic fatigue in remarkably similar terms across these contexts, typically referring to reduced energy, diminished motivation, and impaired engagement in daily activities (Kuppuswamy, 2017, 2022; Raizen et al., 2023). These converging observations—namely, the transversality of intervention efficacy and the striking similarity in symptom perception—pave the way for a novel understanding of chronic fatigue as reflecting the alteration of a shared regulatory mechanism, rather than a condition-specific manifestation Figure 1.

Physical activity and yoga primarily engage bodily and postural systems, whereas CBT and mindfulness predominantly target cognitive and perceptual processes. Despite these differences in their primary domains of action, all interventions converge in promoting active engagement with internal states and environmental demands. This suggests that individual dynamics regulating such bidirectional engagement may be key to treatment response. The heterogeneity of interventions, together with the need to support adherence, calls for the development of personalized pathways that take into account individual attitudes, interests, and specific conditions, including health status as well as social and socioeconomic factors.

### Current research gaps

Three interconnected priorities emerge for future research and clinical steps. First, chronic fatigue requires consistent measurement. Instruments such as the modified Fatigue Impact Scale (mFIS—Fisk et al., 1994) allow standardized monitoring, while future research should aim to integrate stable and easily collectable physiological markers of chronic fatigue. The adoption of shared measures will promote more consistent definitions and terminology, reducing reliance on heterogeneous labels such as Chronic Fatigue Syndrome or pathology-related terms (e.g., cancer-related fatigue). Such advancements are expected to be reflected in future revisions of ICD classifications and diagnostic criteria. The mFIS score may be particularly suitable as a starting point, as normative thresholds have recently been introduced (Strober et al., 2020).

Second, the convergence of effective interventions across conditions supports understanding of chronic fatigue as a key regulatory node in human physiology.

Third, the availability of multiple effective interventions highlights the importance of personalized choice, by identifying the most suitable strategy for each individual, taking into account personal characteristics and contextual factors.

### Potential future developments in the field

**Figure 2:**
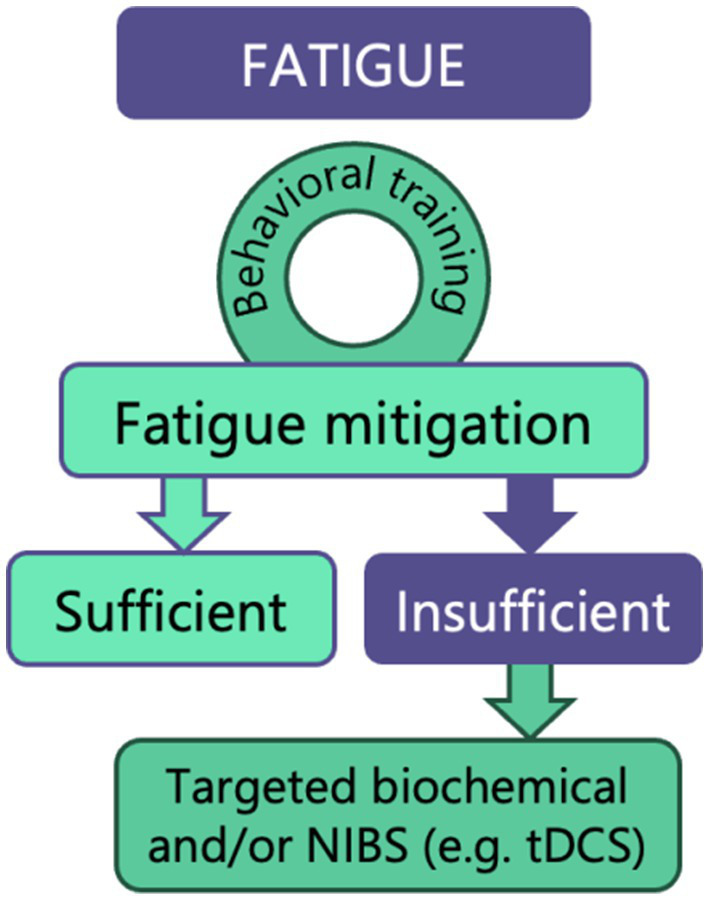
Multimodal fatigue mitigation. Behavioral interventions—the primary strategy to restore regulatory balance through structured movement and cognitive engagement—may, when insufficient, be supported by targeted neurobiological or biochemical interventions and/or non-invasive brain stimulation (NIBS), including transcranial direct current stimulation (tDCS).

When behavioral strategies do not produce sufficient improvement, at least two main explanations may account for this limited efficacy. First, neuronal plasticity underlying learning and adaptation may be constrained by neurobiological and biochemical alterations, limiting the system’s capacity to re-adapt effectively despite appropriate behavioral engagement. This calls for individualized profiling to identify factors hindering recovery and guide targeted interventions (Seidler et al., 2010). Second, altered body–brain communication may impair the integration of sensory information into adaptive decision-making, thereby constraining the effectiveness of behavioral strategies. This perspective is also consistent with evidence from listening-related fatigue, where sustained cognitive effort required to process degraded sensory input has been associated with measurable electrophysiological changes, reinforcing the idea that fatigue may emerge when excessive regulatory effort is required to maintain performance (Strauss et al., 2010). In such cases, non-invasive brain stimulation (NIBS) may support the restoration of functional balance, as suggested by studies in multiple sclerosis showing chronic fatigue reduction following transcranial direct current stimulation (tDCS) (Ayache et al., 2022; Cancelli et al., 2018; Gianni et al., 2021; Tecchio et al., 2014, 2022, 2025; Uygur-Kucukseymen et al., 2023). Within this view, neuromodulation does not replace behavioral training but may facilitate the restoration of functional communication patterns when endogenous regulation is insufficient, thereby enabling adaptive engagement with training processes Figure 2. Notably, multisensory stochastic resonance, especially vibrotactile, has been proposed as a promising novel approach for chronic fatigue, with potential effects in reducing the sensory effort associated with fatigue (White et al., 2019).

## Conclusion

This Mini Review highlights that the same four behavioral interventions consistently mitigate chronic fatigue across heterogeneous conditions, with their transversal impact pointing to a shared underlying regulatory mechanism.

This perspective has relevant implications for both research and clinical practice. It calls for a shift from disease-centered approaches toward a mechanism-oriented framework, where fatigue is considered a key regulatory signal of system imbalance.

Advancing this field will require coordinated efforts across disciplines. The establishment of multidisciplinary consortia may enable the integration of clinical, physiological, and neuro technological knowledge, accelerating the development of effective and scalable strategies for chronic fatigue mitigation.
